# Switching Diagram of Core-Shell FePt/Fe Nanocomposites for Bit Patterned Media

**DOI:** 10.3390/ma15072581

**Published:** 2022-03-31

**Authors:** Yuhui Wang, Ying Zheng, Ziyi Zhong, Zijun Wang, Yongfeng Liang, Pingping Wu

**Affiliations:** 1 Department of Materials Science and Engineering, Xiamen Institute of Technology, Xiamen 361021, Fujian Province, China; yuhuiwang2175@163.com (Y.W.); zhengying_w@126.com (Y.Z.); zzy17321619268@gmail.com (Z.Z.); 2 The Higher Educational Key Laboratory for Flexible Manufacturing Equipment Integration of Fujian Province, Xiamen Institute of Technology, Xiamen 361021, Fujian Province, China; 3 Department of Nuclear Physics, China Institute of Atomic Energy (CIAE), Beijing 102413, Beijing, China; wangzijun@ciae.ac.cn; 4 State Key Laboratory for Advanced Metals and Materials, University of Science and Technology Beijing, Beijing 100083, Beijing, China; liangyf@skl.ustb.edu.cn

**Keywords:** magnetization switching, micromagnetic simulation, core-shell, bit patterned media

## Abstract

In the current work, a core-shell type exchange coupled composite structure was constructed by micromagnetic simulation with a phase FePt core and an iron shell. Four types of switching loops with magnetic domain structure evolution were demonstrated. Based on the simulation results, a switching type diagram was constructed, which displays various hysteresis loops as a function of core radius and shell thickness. Furthermore, the effects of switching type and composite structure on the coercivity and remanent magnetization were predicted and discussed. This finding indicates that core-shell type FePt/Fe composite structure film has a large advantage in designing exchange-coupled bit patterned media to realize high-density storage devices at the nanoscale.

## 1. Introduction

Exchange coupled bit-patterned media are a very promising concept to delay the superparamagnetic size limit for next-generation ultra-high-density magnetic recording storage applications [1,2]. For massive data storage, *L*1_0_ FePt-based thin film/nanodot structure has many advantages, including very large density, low cost, magnetical stability, and chemical stability [3,4]. Therefore, it was considered the most promising candidate for future magnetic recording media material. Beyond this, a composite structure combined with a soft magnetic layer (e.g., an iron phase) creates an exchange coupling structure and helps reverse the magnetization of the hard phase during the writing process with a reduced writing field [5,6,7,8]. This exchange coupling composite (ECC) structure with a sharp/graded interface can further decrease the coercive field and enhance the storage density of the bit patterned media [5,9,10,11].

For traditional double-layered ECC, as a thick, soft layer is introduced, such a composite structure requires more fabrication challenges and large head keeper spacing. Furthermore, it introduces large demagnetization fields and strong dependence on the exchange-coupling strength. Goll et al. proposed several types of ledge-type isolated nanocomposite based on FePt/Fe structure [12]. The investigations show that the coercive field can be significantly decreased compared to the traditional ECC structure [12], which can be experimentally realized by Lomakin et al. [13], Goll et al. [14], and Speliotis et al. recently [15]. In order to further reduce the thickness of composite and keep the advantage of the switching field [16], a structure of enclosed composite patterned media was developed by Goh et al. [17]. Different from the ledge-type structure, the soft layer of the composite is covered all over the surface of the hardcore and creates a core-shell structure in Goh’s work. Their simulation results suggested that this structure provides a lower switching field with lower exchange coupling strength. Following experimental works by Ma et al. [18,19] demonstrate that the switching field, thermal stability, and gain factor of the core-shell structure composite have a large advantage over traditional exchange coupling composite. Some experimental works can present the evolution of the magnetic pattern of composite structure [20,21]; however, the size of the composite is relatively large such as around several hundred nanometers.

It was shown that micromagnetic simulations could predict the magnetic properties for FePt based exchange-coupled bit patterned media [22,23,24,25,26,27]; however, only a few works for core-shell shaped island composite were reported. It should be noted that the hysteresis loop of the core-shell island is very sensitive to the stacking structure of the composite. Our prior works [16,22] on double-layered exchange spring nanoislands demonstrated that the micromagnetic simulations could predict the magnetization hysteresis loop with domain structure evolution at a switching external field. Therefore, the main objective of this letter is to simulate various core-shell type ECC FePt/Fe structures with different core radii and shell thickness by micromagnetic simulation. Four types of switching processes with domain structure evolution were demonstrated in this simulation. The switching diagram was expected to contribute to the design of the core-shell type ECC for bit patterned media by choosing an appropriate core size and shell thickness. The magnetic properties of the core-shelled composite structure were also analyzed and discussed in this work.

## 2. Simulation Method

**Figure 1:**
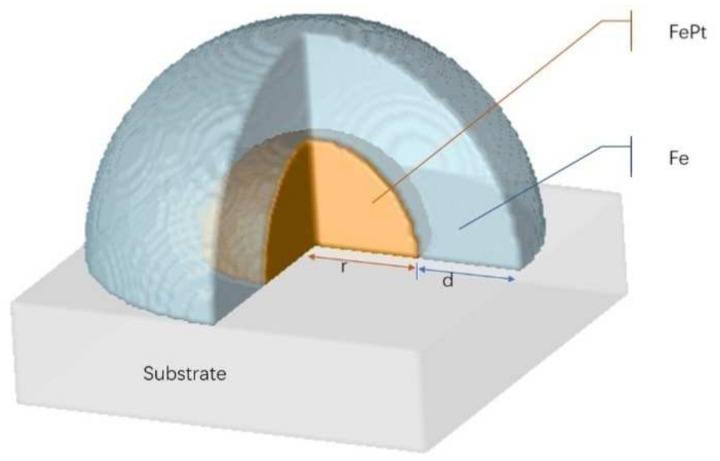
A 3D schematic figure of simulated core-shell structure of FePt/Fe nanocomposite. The FePt core is enveloped by an iron layer.

A core-shell type exchange coupled composite structure was constructed by computer simulation. A hemispherical FePt core island of radius r nm is enveloped by an iron outer layer with a thickness of d nm, as shown in the schematic Figure 1. In our study, a set of FePt/Fe core-shell composite structures was simulated with different core sizes and shell thicknesses. The interfaces between FePt/Fe layers are coherent, and the composite structure is grown epitaxially on a substrate. It should be noted that the interface between the hard and soft magnetic phase was assumed to be a sharp interface in this work; a controlled anisotropy graded interface is not discussed in our current work.

In order to model a proper composite magnetic domain structure by the micromagnetic method, the spontaneous magnetization $M_{i}$(i = 1, 2, 3) were chosen as the order parameter. In micromagnetic simulations, the equilibrium ferromagnetic domain structure can be achieved by solving the Landau–Lifshitz–Gilbert equation, i.e.,

(1) $$\left(1+\alpha^{2}\right)\frac{\partial M}{\partial t}=-\gamma_{0}M\times H_{\mathrm{eff}}-\frac{\gamma_{0^{\alpha }}}{M_{s}}M\times \left(M\times H_{\mathrm{eff}}\right),$$

where α is the damping factor, $\gamma_{0}$ is the gyromagnetic ratio, $M_{s}$ is saturation magnetization, and $H_{\mathrm{eff}}$ is the external magnetic field, i.e.,

(2) $$H_{\mathrm{eff}}=-\frac{1}{\mu_{0}}\frac{\partial E}{\partial M},$$

where $\mu_{0}$ is the vacuum magnetic permeability, E is the total free energy of magnets; in this paper, the total free energy, E, of a FePt/Fe composite is expressed by

(3) $$E=E_{\mathrm{anis}}+E_{\mathrm{exch}}+E_{\mathrm{ms}}+E_{\mathrm{Zeeman}},$$

where $E_{\mathrm{anis}}$, $E_{\mathrm{exch}}$, $E_{\mathrm{ms}}$, and $E_{\mathrm{Zeeman}}$ are magnetocrystalline anisotropy, exchange coupling, magnetostatic, and Zeeman energies, respectively.

The magnetocrystalline anisotropy energy of a cubic magnetic crystal is given by

(4) $$E_{\mathrm{anis}}=\int \left[K_{1}\left(m_{1}^{2}m_{2}^{2}+m_{1}^{2}m_{3}^{2}+m_{2}^{2}m_{3}^{2}\right)+K_{2}m_{1}^{2}m_{2}^{2}m_{3}^{2}\right]\mathrm{dV},$$

where $K_{1}$ and $K_{2}$ are anisotropy constants, $m_{i}=M_{i}/M_{s}$ is unit magnetization, and V is nanostructure volume.

The exchange coupling energy in Equation (3) is determined solely by the spatial variation in the magnetization orientation and can be written as:

(5) $$E_{\mathrm{exch}}=\int {\left(\nabla m\right)}^{2}\mathrm{dV},$$

where A is the exchange coupling constant. In order to calculate the magnetostatics energy of the composite, convolution of magnetization with the three-dimensional LaBonte interaction matrix, the analytic formula developed by Schabes and Aharoni [28] is written in the form

(6) $$E_{\mathrm{ms}}=\frac{1}{2}\sum_{\mathrm{ijk},i\text{′}j\text{′}k\text{′}}^{\text{'}}E_{\mathrm{ijk},i\text{′}j\text{′}k\text{′}},$$

where E_ijk,i′j′k′_ represents the interaction energy between the grids at the site (i, j, k), and the site (i′, j′, k′), (i, j, k) and (i′, j′, k′) are lattice indices in our 3D grids.

The Zeeman energy can be calculated by considering the interactions between magnetization and external magnetic fields $H_{\mathrm{ex}}$:

(7) $$E_{\mathrm{Zeeman}}=-\mu_{0}M_{s}\int H_{\mathrm{ex}}\cdot \mathrm{mdV},$$

In this work, the evolution of the magnetic domain structure and the magnetization distributions are obtained by solving Equations (1)–(7) with the Gauss–Seidel projection method in Ref. [29].

The simulation box of a FePt/Fe composite unit cell is discretized as 32Δx_1_ × 32Δx_2_ × 16Δx_3_, where Δx_1_, Δx_2_, and Δx_3_ are grid spacing, and Δx_1_ = Δx_2_ = Δx_3_ = 1 nm for real space. In this work, the radius of the FePt core changes from 3 nm to 10 nm, the nominal thickness of the iron layer changes from 3 nm to 7 nm, and the shell thickness are similar to that of experimental works reported by Ma et al. [18]. All the corresponding magnetic coefficients, including magnetization saturation, anisotropy constant, exchange coupling constant, were taken from experimental data from Ref. [30] and listed as follows: for the FePt core: $M_{s}$ = 1.050 × 10^6^ A/m, $K_{1}$ = 1.7 × 10^6^ J/m^3^, $K_{2}$ = 0 J/m^3^, A = 10^−11^ J/m; for the Fe shell: $M_{s}$ = 1.575 × 10^6^ A/m, $K_{1}$ = 4.7 × 10^4^ J/m^3^, $K_{2}$ = 0 J/m^3^, A = 2 × 10^−11^ J/m. In this work, the micromagnetic simulation is performed using GNU Octave, and the code was programmed by the author.

## 3. Results and Discussion

**Figure 2:**
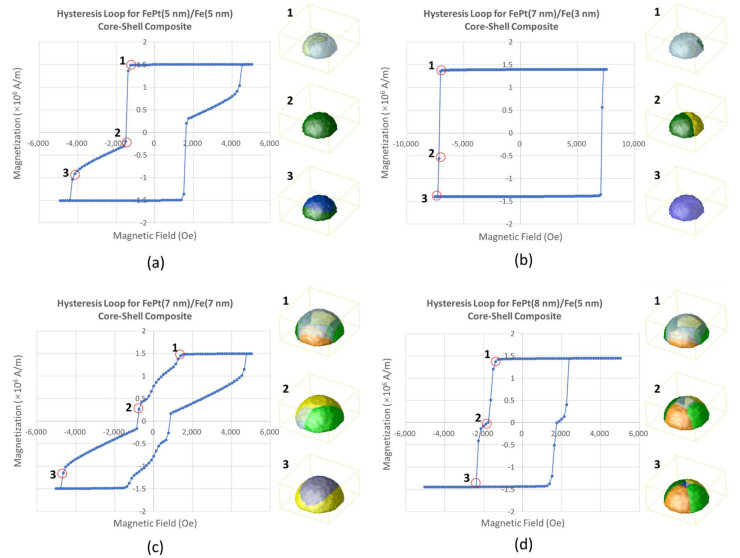
(**a**–**d**) Magnetic hysteresis loops of FePt/Fe core-shelled structure measured in the out-of-plane directions for the switching type of A–D, respectively. The simulated corresponding magnetic domain structure evolution during the switching process at the position of three circles 1, 2, 3 are shown at the right of the loop. Each color represents a type of ferromagnetic domain: yellow/orange represents +x/−x domains, green/dark green represents +y/−y domains, and blue/light blue represents +z/−z domains.

In order to simulate a switching process of the composite island, the initial domain structure was magnetized to saturation alongx direction, and then we decreased the external field by a step of 10 kA/m (~125 Oe) and finally reversed the field direction to saturate magnetization in the opposite direction. In our simulation, we found that different simulation loops could be obtained according to the different core radii and shell thickness; at this point, we found four representative switching loops. Examples of the switching loop with the type of A–D and the magnetic domain evolution are shown in Figure 2a–d, respectively. For type A, a constricted hysteresis loop was obtained in FePt(5 nm)/Fe(5 nm) core-shell type composites. The magnetization sharply decreased to zero at a negative field, then slowly switched to the opposite direction. For type B, as the hard material FePt increases to 7 nm while Fe decreases to 3 nm, the simulation results of the composite material show a square hysteresis loop; however, while the soft layer increases to 7 nm, a type C switching loop shows a narrow hysteresis with small amounts of residual magnetism and coercive field. For type D, a typical exchange-coupled switching loop for a FePt(8 nm)/Fe(5 nm) composite was observed, resulting in a smaller coercivity and saturation field.

In order to explore the influence of the ferromagnetic domain structures on the switching loops, we analyzed the effect of ferromagnetic domain structures evolution for different switching types, which are illustrated in the right column of Figure 2. The light blue/blue domains represent the domains with magnetization towards +z/−z directions, i.e., external field directions. The yellow/orange/green/dark green domains represent the in-plane domains with magnetizations along with +x/−x/+y/−y directions, respectively.

According to classical magnetic theory [31], one can estimate the size of the particles by balancing the energy needed to create a domain wall spanning a spherical particle and the magnetostatic energy saved by reducing the single domain state to a multi-domain state; thus, this method gives the critical radius of the particle *r*_c_(Fe) ~3 nm and *r*_c_(FePt) ~27 nm. According to Kikuchi et al., experimental report [32], the critical diameter of the single domain FePt particles is around 55 nm, which is consistent with the theory. In this simulation work, the FePt core is always observed to stay in a single domain state because the radius of the FePt core is much less than the critical radius; on the other hand, the thickness of the iron shell is larger than 3 nm, which exceed the predicted critical value; therefore, the iron shell always exhibits a more stable multi-domain state in our simulation.

In the FePt(5 nm)/Fe(5 nm) core-shell composites, the magnetization quickly decreased to below zero at −1500 Oe. In the domain structures, one can see both the soft shell and hardcore are switched to in-plane direction. In the next stage, the magnetization in the soft magnetic phase was gradually switched to −z-direction and resulted in a slight decrease in magnetization. Finally, the whole structure was switched to the −z-direction with a jump of magnetization led by the switching of the hard FePt core, and, as a result, the magnetization reached full saturation in the opposite direction, and a constricted loop (type A) was constructed.

A FePt(7 nm)/Fe(3 nm) composite exhibited a square-like hysteresis loop (type B), which is analogous to the loops of hard magnets. A sharp decrease in magnetization was observed at around −570 kA/m (~−7160 Oe). We observed that the magnetization of the whole structure rotates to the in-plane direction quite rapidly, then to the −z-direction, resulting in a high remanent magnetization and a large coercive field. In contrast, if we kept the size of the FePt core and increased the thickness of the iron shell to 7 nm, a switching loop similar to that of soft magnets (type C) was observed in our simulation. The magnetization in the iron shell phase started to rotate before the external field was completely withdrawn, whereas the 90-degree domain wall motion was taking place, leading to a decrease in magnetization. Note that a small jump of magnetization was observed at saturation in the opposite direction, which can be attributed to the switching of the hard magnetic FePt core.

For a composite of FePt(8 nm)/Fe(5 nm) structure, a hysteresis loop exhibited a two-step process (type D) with a small coercive field but a large remanent magnetization, which is typical for “exchange-coupled” magnetic composite. Interestingly, a vortex structure formed in the soft magnetic shell as a nucleus, further decreasing the external field; the formed nucleus propagated to the soft/hard interface where the magnetization became pinned. As the soft shell is strongly exchanged coupled to the hardcore, the whole structure is completely reversed to the opposite direction. The simulation results show that in this case, there is a presence of an exchange coupling effect, and the reduction in coercivity is much more significant, which is very close to the theoretical predictions of Goh et al. [17].

**Figure 3:**
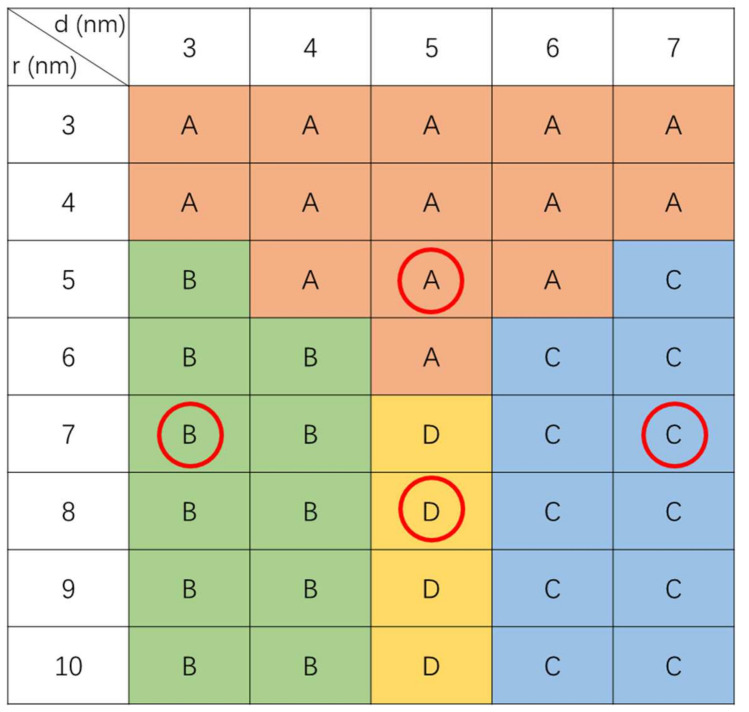
The switching type diagram of FePt/Fe core-shelled structure as a function of the thickness of the iron shell and the radius of FePt core. d and r in the figure represents the thickness of iron shell in the range of 3–7 nm and the radius of FePt core in the range of 3–10 nm, respectively. The ○ indicates the location of the switching loops shown in Figure 2. The symbols A, B, C, and D represent the switching type A, B, C, and D of the core-shelled FePt/Fe structure in Figure 2, respectively.

Based on the simulation results, a switching type diagram, i.e., a representation of switching loops as a function of core radius and shell thickness, is constructed and illustrated in Figure 3. The radius of the FePt core changes from 3 nm to 11 nm, and the nominal thickness of Fe varies from 3 nm to 7 nm. In the diagram, if the FePt core size is small, the switching hysteresis exhibits a constricted loop (type A); this behavior is due to the hardcore as a small single-domain particle is easy to be switched. For large FePt core size, the switching type is sensitive to the thickness of the iron layer. The switching diagrams indicate that for a relatively large core size (r > 6 nm), there are three different types of switching loops with increasing iron layer thickness from 3 nm to 7 nm. If the shell thickness is lower than 4 nm, the switching mechanism is dominated by the FePt core. The hysteresis loop exhibits a square-like switching loop (type B), which is analogous to the loops of hard magnets. In contrast, when the iron layer thickness increases to 6 nm, the magnetization in the soft layer starts to rotate before the external field is reduced to zero, which results in a decrease in remanent magnetization. Further increasing the iron layer thickness, the calculated hysteresis loop is analogous to the loops of soft magnets (type C), as the iron layer plays a major role in the switching process. It should be noted that a transition region can be observed between type B and type C regions, as can be seen in Figure 3. By controlling the thickness of the iron shell to ~5 nm, a hysteresis loop with relatively high remanent magnetization and a small coercive field is confirmed, which can be attributed to a typical exchange-coupled effect in the composite magnets.

**Figure 4:**
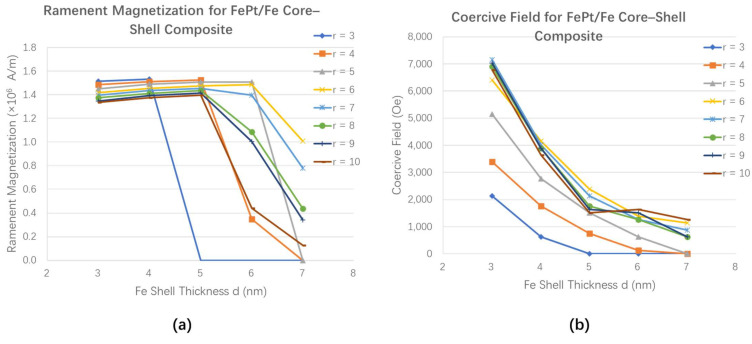
The magnetic properties as a function of the thickness of the iron shelland the radius of FePt core. (**a**) The remanent magnetization (**b**) the coercivities. In this figure, r is the radius of FePt core.

In order to probe the expected influence of the composite structures on the magnetic properties, the calculated remanent magnetization and the coercive field are summarized in Figure 4. The remanent magnetization and coercive field can be tuned by changing the core size and soft layer thickness of the composite. Obviously, the remanent magnetization and coercive field decrease with the thickness increase in the iron layer. In contrast, increasing the hardcore radius can increase the coercive field of the composite. It is also interesting to note that for the composites with the same shell thickness, the coercive field has minor changes with the radius of FePt core when the core radius is beyond 6 nm. This phenomenon is because the increase in hardcore radius also increases the volume of the soft layer. In general, the magnetization properties of the composite are strongly influenced by the switching type of the hysteresis loops.

**Figure 5:**
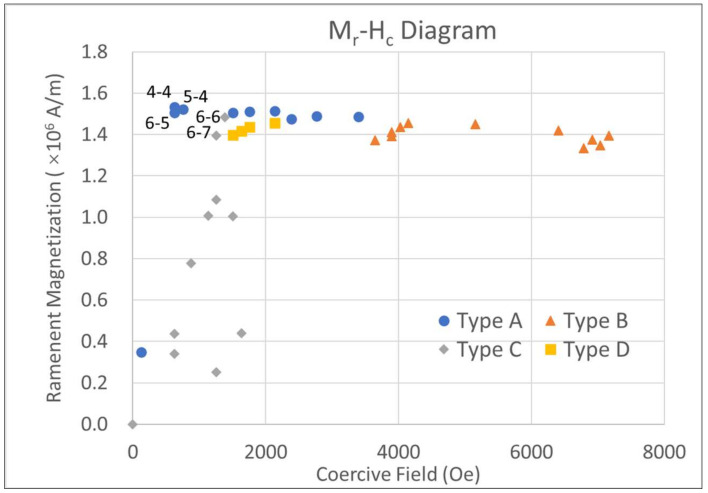
The remanent magnetization-coercive field (M_r_–H_c_) diagram for the core-shell FePt/Fe composite. Solid circles, triangles, diamonds, and squares denote the M_r_ and H_c_ of loops of type A–D in Figure 3, respectively. Some key structures with their geometric structure (FePt radius-Fe thickness, in the unit of nm, e.g., 4–4 represents a composite structure with a core of 4 nm radius and a shell of 4 nm thickness) are also noted in the diagram.

For achieving high-density memory devices, bit patterned media with a lower coercive field, lower anisotropy field, smaller island sizes, and a high remanence is required [3,9,15,19]. Hereby, a remanent magnetization-coercive field (M_r_–H_c_) diagram was introduced to illustrate the shape of the hysteresis loops, as shown in Figure 5. It is easy to see the typical hard magnets with high coercive field and high remanence located in the upper right corner of the M_r_–H_c_ diagram, while typical soft magnets with low coercive field and low remanence are located in the lower-left corner of this diagram. The magnetic composite with the geometric structures located in the upper left corner of Figure 5 is our aim material, which meets the requirements for high-density storage devices. As expected, type B hysteresis loops exhibit high remanent magnetization and high coercive field, while type C loops show a hysteresis similar to soft magnetic materials with relatively small remanent magnetization and small coercive field. Respectively, in the M_r_–H_c_ diagram, the magnetic composites of type B and type C are distributed in the upper right and lower left corners of the diagram in a relatively large range. Thus, both type B and type C hysteresis loops are not suitable for the bit patterned memory devices. The candidate structure for bit patterned media should have a hysteresis loop of type A or type D. As shown in Figure 5, the magnetic properties of composites of type A and type D are concentrated in the upper left corner of the diagram with a relatively small range, which means that the exchange coupling effect of type A/type D loops can lead to the smaller coercive field and maintaining high remanent magnetization. If considering the size of the composite island, the type A hysteresis loop with a core radius around 3–4 nm and a shell thickness of 3–5 nm may potentially find uses in future memory devices. A disadvantage of the type A hysteresis loop is that a relatively high anisotropy field is observed, which results in a higher switching field and may limit its applications. Therefore, further studies are necessary to decrease the anisotropy field of the type A hysteresis loop.

## 4. Conclusions

In this work, a switching type diagram as a function of the iron shell thickness and the FePt core radius was constructed for FePt/Fe core-shell composite structure based on a micromagnetic simulation. Four types of switching types were found in the core-shell type composite with different core sizes and shell thicknesses. This study demonstrated that the magnetic switching hysteresis loop could be tuned by controlling the core size and the shell thickness of the core-shell composite. Furthermore, the magnetic domain configuration during the switching process was analyzed to obtain insight into the mechanisms of the switching properties. The remanent magnetization and the coercive field for the core-shell type composite were predicted based on the switching type diagram. The possible composite structure for bit patterned media should have a hysteresis of type A or type D because the exchange coupling effect between the hard magnetic core and soft iron shell lowered the coercive field but maintained high remanent magnetization. These simulation results should motivate further exploration of magnetic composite structures with smaller sizes and better performance, and it will be beneficial in future design and development of bit patterned memory device applications.

## Data Availability

Not applicable.
